# The interaction between social factors and adversities on self-harm during the COVID-19 pandemic: longitudinal analysis of 49 227 UK adults

**DOI:** 10.1192/bjo.2021.1071

**Published:** 2021-12-10

**Authors:** Elise Paul, Daisy Fancourt

**Affiliations:** Department of Behavioural Science and Health, University College London, UK

**Keywords:** Self-harm behaviours, longitudinal studies, COVID-19, self-harm thoughts, adversity

## Abstract

**Background:**

Little is known about which factors exacerbate and buffer the impact of coronavirus disease 2019 (COVID-19)-related adversities on changes in thinking about and engaging in self-harm over time.

**Aims:**

To examine how changes in four social factors contribute to changes in self-harm thoughts and behaviours over time and how these factors in turn interact with adversities and worries about adversities to increase risk for these outcomes.

**Method:**

Data from 49 227 UK adults in the UCL COVID-19 Social Study were analysed across the first 59 weeks of the pandemic. Fixed-effects logistic regressions examined time-varying associations between social support quality, loneliness, number of days of face-to-face contact for >15 min and number of days phoning/video calling for ≥15 min with self-harm thoughts and behaviours. We then examined how these four factors in turn interacted with the total number of adversities and worries about adversity and how this affected outcomes.

**Results:**

Increases in the quality of social support were associated with decreases in the likelihood of both outcomes, whereas greater loneliness was associated with an increase in their likelihood. Associations were less clear for telephone/video contact and face-to-face contact with outcomes. Social support buffered and loneliness exacerbated the impact of adversity experiences on self-harm behaviours.

**Conclusions:**

These findings suggest the importance of the quality of one's social support network, rather than the mere presence of contact, for reducing the likelihood of self-harm behaviours in the context of COVID-19 pandemic-related adversity and worry.

## Background

There is concern that the coronavirus disease 2019 (COVID-19) pandemic and its associated stressors will result in an increase in suicides, although this is not inevitable.^1,2^ This is hypothesised to occur via reductions in protective factors such as social connectedness and increases in risk factors such as domestic misuse and unemployment.^2–4^ According to diathesis–stress models of suicide risk, the experience of these near-term stressors for individuals who already have enduring risk factors (such as traits of impulsivity, genetic vulnerability and having experienced adversity early in life) could lead to an increase in self-harm behaviours and ultimately suicide.^5–7^

There is evidence suggesting that the pandemic has had a detrimental effect on self-harm thoughts and behaviours, that significantly increase the probability of eventual death by suicide.^8,9^ Although early in the pandemic in the UK and France there was a reduction in the number of clinical presentations for self-harm compared with prior years, this could have been because of a decrease in face-to-face services and a wish to protect healthcare services.^10–12^ Data from electronic general practice records in a large UK city through to the end of May 2021 indicate this trend in self-harm presentations may have persisted after restrictions eased.^13^ However, survey data^14–16^ and a recent systematic review^17^ suggest that a greater proportion of the population have been thinking about or actually harming themselves than pre-pandemic. Stressful life events, which can precipitate self-harm thoughts and behaviours in the short term such as domestic misuse^18^ and widespread unemployment,^19,20^ have also increased. Concerns and worries about these and other adverse events that can be proximal triggers for self-harm^6,7^ have been found to have a similar impact on self-harm thoughts and behaviours as actually experiencing these adversities during the COVID-19 pandemic.^21^ Further, the combination of the unprecedented social distancing requirements, uncertainty about the future and the accumulation of stressful circumstances have the potential to increase risk for suicides.^2,3^

There are, however, factors that could protect against the impact of these stressful circumstances on self-harm thoughts and behaviours. More frequent contact with others as well as the quality of one's social support has been shown to provide a buffer against the likelihood of self-harm in the context of acute stressors such as financial difficulties or relationship breakdown.^22,23^ Given that the most commonly cited reasons for self-harm are to relieve suffering and manage distress,^24,25^ it follows that having access to supportive, understanding others would help mitigate the adverse consequences occurring in the context of the pandemic. However, social restrictions imposed during the pandemic may have severely limited access to drawing on and maintaining connections vital to reducing the impact of this stress. Therefore, an unresolved question is whether the perceived quality of one's social support and more frequent contact with others buffer the impact of adversity and worry about adversity on thinking about and engaging in self-harm.

A second factor that may be important for the link between pandemic-related adversities and self-harm thoughts and behaviours is loneliness,^2^ or the subjective distress resulting from a discrepancy between desired and perceived social relationships.^26^ Loneliness and a lack of social integration have been emphasised as important factors for suicide from Durkheim's early sociological studies of suicide^27^ to modern theories of suicide risk.^6,7^ Although early in the pandemic loneliness did not seem to be higher than pre-pandemic levels,^22,28^ data from the Office of National Statistics’ Opinions and Lifestyle Survey in the UK indicate that over the first year of the pandemic, the proportion of people who were lonely ‘often’ or ‘always’ increased (5.0% to 7.2%).^29^ Thus, risk for self-harm thoughts and behaviours because of adversities and worries about adversity may have been exacerbated by increased levels of loneliness.

## Aims

In sum, although the COVID-19 pandemic has had a detrimental impact on a number of known risk factors for suicide, little is known about how social factors such as social support, social contact and loneliness may have interacted with these risk factors to exacerbate or buffer their impact on self-harm thoughts and behaviours. The aim of this study was therefore to examine which near-term social factors were associated with changes over time in self-harm thoughts and behaviours in a large sample of UK adults across the first 59 weeks of the COVID-19 pandemic. Specifically, we explored the time-varying longitudinal relationships between (a) the quality of one's social support, (b) loneliness, (c) time spent in face-to-face contact with others, and (d) time spent in phone or video contact with others with changes in these two outcomes. We then also examined whether these factors moderated the relationship between experiencing and worrying about adversities and self-harm thoughts and behaviours. We improve upon prior research in this area by using fixed-effects statistical modelling^30^ that automatically accounts for longer-term more stable risk factors for self-harm thoughts and behaviours such as genetic predisposition and certain personality traits.^5,7^ The findings will further our understanding of which factors attenuate and exacerbate the risk for self-harm thoughts and behaviours in the context of the COVID-19 pandemic, which is important for informing suicide prevention efforts.

## Method

### Participants

We used data from the COVID-19 Social Study; a large panel study of the psychological and social experiences of over 70 000 adults (aged 18 or older) in the UK during the COVID-19 pandemic. The study commenced on 21 March 2020 and involved online weekly (from August 2020, monthly) data collection across the pandemic. Sampling was not random, and although it is heterogeneous, the sample is not representative of the UK population. The sample was recruited using three primary approaches. First, convenience sampling was used, including promoting the study through existing networks and mailing lists (including large databases of adults who had previously consented to be involved in health research across the UK), print and digital media coverage, and social media. Second, more targeted recruitment was undertaken focusing on (a) individuals from a low-income background, (b) individuals with no or few educational qualifications, and (d) individuals who were unemployed. Third, the study was promoted via partnerships with third-sector organisations (for example charities or community sector organisations) to include marginalised or vulnerable groups including adults with pre-existing mental health conditions, older adults, carers and people experiencing domestic violence or abuse.

The authors assert that all procedures contributing to this work comply with the ethical standards of the relevant national and institutional committees on human experimentation and with the Helsinki Declaration of 1975, as revised in 2008. The study was approved by the UCL Research Ethics Committee (12467/005) and all participants gave informed consent. The study protocol and user guide (which includes full details on recruitment, retention, data cleaning and sample demographics) are available at https://github.com/UCL-BSH/CSSUserGuide.

For these analyses, we used data from the 14 months between 1 April 2020 to 17 May 2021 (participant *n* = 66 308, observation *n* = 918 440). Participants were eligible for inclusion in the analysis if they had three or more data collections over the study period (participant *n* = 52 569 (79.3%), observations = 899 447 (97.9%)). We excluded participants with missing data on any of the variables in the study. The final sample size was 49 227 (observation *n* = 849 800). See Supplementary Table 1 available at https://doi.org/10.1192/bjo.2021.1071 for a comparison of excluded and included participants.

### Measures

#### Self-harm thoughts and behaviours

The definition of self-harm used in the current study was intentional non-fatal acts of self-poisoning or self-injury, irrespective of the degree of suicidal intent.^31,32^ Thoughts of death or self-harm (hereafter self-harm thoughts) were measured with an item from the Patient Health Questionnaire (PHQ-9);^33^ an instrument often used as a screening tool for depression in primary care practice: ‘Over the last week, how often have you been bothered by thoughts that you would be better off dead or hurting yourself in some way?'. Second, self-harm behaviours were measured with a similar study-developed item: ‘Over the last week, how often have you been bothered by self-harming or deliberately hurting yourself?'. Responses to both items were rated on a four-point scale from ‘not at all' to ‘nearly every day' and collapsed into binary variables indicating the presence of at least some self-harm thoughts or self-harm behaviours at each time point.

### Social support

#### Perceived social support

Social support in the past week was measured using an adapted version of the six-item short form of Perceived Social Support Questionnaire (F-SozU K-6).^34,35^ Each item is rated on a five-point scale from ‘not true at all' to ‘very true', with higher scores indicating higher levels of perceived total social support (hereafter, ‘social support’). Minor adaptations made to question phrasing to make it relevant to experiences during COVID-19 can be found in Supplemental Table 2. Mean social support scores were calculated at each time point (range 1–5). As a sensitivity analysis, we disaggregated the total social support variable into emotional support and instrumental support (three items each) to examine whether the provision of instrumental assistance or emotional support may have been driving any findings.

#### Time spent in contact with others

Two continuous variables representing the number of days participants (a) had face-to-face contact with another person for 15 min or more (including someone the participant lives with), and (b) had a phone or video call with another person for 15 min or more in the past week were included. The mean for each of these variables was calculated at each time point (range 0–7).

#### Loneliness

Loneliness was measured using the three-item UCLA-3 Loneliness, a short form of the Revised UCLA Loneliness Scale (UCLA-R).^36^ Each item is rated with a three-point rating scale, ranging from ‘hardly ever' to ‘often'. The mean of these three items was calculated for each participant and higher scores indicate higher loneliness (range 1–3).

### Adversity experiences and worries

#### Adversity experiences

Five categories of adversities occurring in the past week were considered: financial adversity, COVID-19 illness, family/friend serious illness or bereavement, experiencing physical or psychological abuse, and not being able to access essential items. Each category of adversity was treated as binary (absent versus present) and summed to create an index of the number of adversities experienced at each time point (range 0–5). A more detailed description of these measures can be found in Supplementary Table 3.

#### Worries about adversity

Five worries about adverse experiences were measured at the same time as the adversity measures and selected to correspond with these variables. Each category of worry was operationalised as binary (absent versus present): financial worries, worries about COVID-19 illness, social and relationship worries, concerns about safety and security, and worries about accessing essentials. These binary variables were then summed to create the total number of worries about adversity at each time point (range 0–5).

### Statistical analysis

We used fixed-effects regression to explore the time-varying relationship between changes in social support, time spent in contact with others face-to-face and via telephone/video, and loneliness with changes in self-harm thoughts and behaviours over the course of the study period (1 April 2020 to 17 May 2021). Participants completed the weekly questions an average of 17.28 (s.d. = 9.60) times during this time (range 3–31), providing longitudinal data to analyse. Fixed-effects regression differs from other regression techniques as it explores within-person variation with individuals serving as their own reference point, compared with themselves over time. As a result, fixed-effects regression accounts for any confounding associations between time-invariant (stable) covariates, even if they are unobserved in the data-set. Such time-invariant covariates include socioeconomic status, genetics, personality and history of mental health problems.^37^ So in this analysis we were able to assess the relationship between changes in social factors across the 59 weeks and changes in self-harm over the same period, while accounting for all possible time-invariant confounders. See the Supplementary materials for more detail, including the model equation.

We then repeated these models additionally controlling for the effects of time-varying confounders including the total number of adversities and the total number of worries about adversity that people experienced at each weekly time point, as well as the interactions between these variables and each of the four social variables in turn. Models additionally controlled for day of the week and number of days since the first UK lockdown commenced. Resulting regression coefficients were exponentiated and presented as odds ratios along with 95% confidence intervals.

To account for the non-random nature of the sample and to increase the representativeness of the UK general population, all data were weighted to the proportions of gender, age, ethnicity, country and education obtained from the Office for National Statistics.^38^ Weights were constructed using a multivariate reweighting method using the Stata user written command ‘ebalance’.^39^ Analyses were conducted using Stata version 16.^40^

Sensitivity analyses with the social support variable disaggregated into emotional support and instrumental support (three items each) were conducted to examine whether the provision of instrumental assistance or emotional support was driving any social support findings.

## Results

### Descriptive statistics

Descriptive statistics for the total sample and for those with any change over the study period in self-harm thoughts or self-harm behaviours are presented in Supplementary Table 4. Before weighting, the total sample was disproportionately female, of older age and highly educated. After weighting, sample proportions reflected those of the UK population. The average number of measurement points in the total sample was 17.28 (s.d. = 9.60, range 3–31) There was within-individual variation in both of the self-harm outcome measures, predictors and adversity measures (Table 1). Nearly one-quarter (23.5%) of the sample reported self-harm thoughts at least once over the study period, and 7.6% reported self-harm behaviours at least once.

**Table 1:** Means and standard deviations for study outcomes and exposures among individuals with variation in each outcome variable (n = 49 227)

	Participants with variation in self-harm thoughts						Participants with variation in self-harm behaviours					
Variable	Overall mean	Overall s.d.	Between s.d.	Within s.d.	*n*	Mean (s.d.) range	Overall mean	Overall s.d.	Between s.d.	Within s.d.	*n*	Mean (s.d.) range
Observations	–	–	–	–	206 437	–	–	–	–	–	63 632	–
Individuals	–	–	–	–	11 559	–	–	–	–	–	3 740	–
Self-harm thoughts	0.28	0.45	0.26	0.36	–	–	0.47	0.50	0.36	0.34	–	–
Self-harm behaviours	0.04	0.20	0.13	0.16	–	–	0.20	0.40	0.23	0.33	–	–
Adversity experiences	1.37	0.61	0.49	0.41	–	–	1.43	0.67	0.55	0.44	–	–
Adversity worries	2.18	1.21	0.96	0.77	–	–	2.37	1.28	1.02	0.80	–	–
Social support	3.31	1.18	1.07	0.44	–	–	3.06	1.23	1.10	0.46	–	–
Emotional support	3.31	1.26	1.11	0.53	–	–	3.07	1.30	1.14	0.55	–	–
Instrumental support	3.30	1.26	1.14	0.49	–	–	3.04	1.31	1.18	0.51	–	–
Loneliness	1.98	0.68	0.60	0.32	–	–	2.15	0.68	0.60	0.32	–	–
Face-to-face contact	5.35	2.58	2.15	1.41	–	–	5.13	2.66	2.22	1.49	–	–
Telephone/video contact	3.30	2.31	1.88	1.37	–	–	3.08	2.31	1.87	1.40	–	–
Number of time points	–	–	–	–	–	18.26 (9.08) 3–31	–	–	–	–	–	17.44 (9.15) 3–31

### Associations between predictor variables and self-harm thoughts and behaviours

Better quality social support was associated with the largest reductions in the odds of self-harm thoughts (odds ratio (OR) = 0.55, 95% CI 0.54–0.57) and self-harm behaviours (OR = 0.71, 95% CI 0.68–0.74), whereas loneliness was associated with a 3.77 (95% CI 3.61–3.93) times higher odds of self-harm thoughts and 2.18 (95% CI 2.02–2.34) higher odds of self-harm behaviours (Table 2). The number of days on which individuals had had face-to-face contact or telephone/video contact with another person for at least 15 min were associated with small reductions and increases in self-harm thoughts, respectively, but was not associated with self-harm behaviours.

**Table 2:** Associations between predictor variables with self-harm thoughts and behaviours (main effects) derived from fixed-effects logistic regression models^a^

	Self-harm thoughts		Self-harm behaviours	
Variable	OR	95% CI	OR	95% CI
Social support	0.55*	0.54–0.57	0.71*	0.68–0.74
Loneliness	3.77*	3.61–3.93	2.18*	2.02–2.34
Face-to-face contact	1.01*	1.00–1.01	1.00	0.98–1.01
Telephone/video contact	0.98*	0.97–0.99	1.01	0.99–1.03

a. Data were weighted to the proportions of gender, age, ethnicity, country and education obtained from the Office for National Statistics.

* *P* < 0.05.

### Interactions between predictors and adversities and adversity worries

Associations between predictor variables, adversities and adversity worries, as well as their interactions with outcomes are shown in Table 3. Main associations between adversity worries and both outcomes were generally larger in magnitude (OR range 1.24–1.42) than for actual adversity experiences (OR range 1.08–1.14). Social support and loneliness continued to be associated with reduced and increased likelihood, respectively, of self-harm thoughts and behaviours, even when adversities and worries were included in the models.

**Table 3:** Associations between adversity experiences and adversity worries with self-harm thoughts and behaviours (main effects and interaction terms with predictor variables) derived from fixed-effects logistic regression models

	Self-harm thoughts		Self-harm behaviours	
	OR	95% CI	OR	95% CI
Adversity experiences				
Adversity experiences	1.13*	1.11–1.15	1.10*	1.06–1.13
Social support	0.42*	0.40–0.43	0.63*	0.60–0.66
Interaction with social support	1.00	0.99–1.02	0.97*	0.95–0.99
Adversity experiences	1.13*	1.11–1.15	1.08*	1.04–1.12
Loneliness	2.62*	2.55–2.69	1.72*	1.64–1.80
Interaction with loneliness	0.99	0.97–1.00	1.04*	1.01–1.06
Adversity experiences	1.13*	1.12–1.15	1.13*	1.10–1.16
Face-to-face contact	0.94*	0.92–0.96	0.96*	0.92–0.99
Interaction with face-to-face contact	0.99	0.98–1.00	1.00	0.99–1.02
Adversity experiences	1.14*	1.12–1.15	1.13*	1.10–1.16
Telephone/video contact	0.91*	0.89–0.93	0.99	0.95–1.02
Interaction with telephone/video contact	0.98	0.97–1.00	1.01	0.99–1.03
Adversity worries				
Adversity worries	1.40*	1.37–1.43	1.31*	1.25–1.36
Social support	0.42*	0.41–0.44	0.62*	0.59–0.66
Interaction with social support	1.01	0.99–1.02	1.03*	1.00–1.06
Adversity worries	1.40*	1.36–1.43	1.24*	1.19–1.31
Loneliness	2.60*	2.52–2.67	1.69*	1.61–1.78
Interaction with loneliness	0.95*	0.94–0.97	1.00	0.97–1.03
Adversity worries	1.42*	1.39–1.45	1.30*	1.26–1.35
Face-to-face contact	0.93*	0.91–0.95	0.95*	0.92–0.99
Interaction with face-to-face contact	1.02*	1.00–1.03	1.02	0.99–1.04
Adversity worries	1.42*	1.39–1.45	1.31*	1.27–1.36
Telephone/video contact	0.90*	0.88–0.92	0.96	0.92–1.00
Interaction with telephone/video contact	0.98*	0.96–0.99	1.04*	1.02–1.07

* *P* < 0.05.

There was evidence that the relationship between adversity worries and self-harm thoughts was slightly attenuated by lower levels of loneliness (OR = 0.95; 95% CI 0.94–0.97) and increases in days of telephone/video contact (OR = 0.98; 95% CI 0.96–0.99), but this relationship was slightly exacerbated by days of face-to-face contact (OR = 1.02; 95% CI 1.00–1.03).

For self-harm behaviours, loneliness exacerbated (OR = 1.04; 95% CI 1.01–1.06) the association of adversity experiences with this outcome, whereas better quality social support attenuated this association (OR = 0.97, 95% CI 0.95–0.99). Social support and days of telephone/video contact exacerbated the relationship between adversity worries and self-harm behaviours.

### Sensitivity analyses

Sensitivity analyses with the disaggregated social support scale into emotional and instrumental support indicated slightly stronger associations between emotional support and self-harm thoughts (OR = 0.53; 95% CI  0.51–0.55) than between instrumental support and self-harm thoughts (OR = 0.76; 95% CI 0.74–0.79) (Supplementary Table 5). However, the differences between these two types of support were negligible in relation to self-harm behaviours. Other substantive findings for the main associations of adversities and adversity worries were the same as in the main analyses (Supplementary Table 6). There was weak evidence that emotional support exacerbated the relationship between adversity worries and both outcomes. Instrumental support was the only variable to attenuate any of the associations (adversity experiences with self-harm behaviours).

## Discussion

### Main findings

Better quality social support was associated with a considerably reduced likelihood of both self-harm thoughts and self-harm behaviours across the first 59 weeks of the COVID-19 pandemic. Having had more days of telephone/video contact with another person for 15 min or more was associated with only minor reductions in the likelihood of self-harm thoughts and was not associated with changes in self-harm behaviours. Additionally, increases in loneliness were associated with a nearly 4-fold and over 2-fold likelihood of self-harm thoughts and behaviours, respectively. These findings suggest that the quality of social interactions rather than the mere presence or absence of social contact are important for these outcomes in the current circumstances.^3^

In support of this, better quality social support acted as a moderator of the impact of adversity experiences on self-harm behaviours, which echoes research from before the current pandemic.^22,23^ Further, higher levels of loneliness exacerbated the impact of adversity experiences on self-harm behaviours. It is notable that the associations were specifically with self-harm behaviours; neither social support nor loneliness buffered the relationship between adversity experiences and thoughts about self-harm. Loneliness, low social support and adversities such as unemployment and financial problems are known risk factors for self-harm and also for suicide, and the findings presented here confirm predictions from early in the COVID-19 pandemic that they would combine and exacerbate one another.^1,41^ Although in the current study this attenuation was modest, these results suggest the importance of available trusted others to provide understanding and support during pandemics, especially for individuals experiencing stressful life events.

Worries about adversity were more strongly associated with both outcomes than actual adversity experiences, a finding that echoes previous research on self-harm thoughts and behaviours and symptoms of anxiety and depression.^21,42^ Unexpectedly, however, the interactions between social support and loneliness, and adversity worries and self-harm thoughts and behaviours, respectively, were in the opposite direction. It is possible that people with higher levels of social support and lower loneliness talked about their worries more with others, with such conversations possibly leading to their worries being more prominent in their minds when they were then asked to self-report on them. This theory is supported by the fact that face-to-face contact exacerbated the relationship between adversity worries and self-harm thoughts, and telephone/video contact also exacerbated the relationship between adversity worries and self-harm behaviours. Nonetheless, telephone/video calls still buffered the relationship between both adversity worries and experiences and self-harm thoughts. Future research could seek to disentangle whether the nature of telephone/video contact affects this moderation effect: it remains unclear whether these calls were made to friends/family, work colleagues or telephone helplines such as the Samaritans. Prior findings using data from the same study as in the current analyses found that in the first month of the pandemic, small proportions of people who had reported self-harm thoughts (2.1%) or self-harm behaviours (4.6%) had utilised a helpline for mental health support, and around one-third had spoken with a friend or family member about their mental health.^15^

Analyses that disaggregated the social support measure into emotional and instrumental forms of support indicated that the quality of one's emotional support, such as experiencing a lot of understanding and help from others and someone to talk to when feeling down, may be more important for self-harm thoughts than having someone to borrow something from or spend time with (instrumental support). However, there was evidence that instrumental support (not emotional support) buffered the association between experiencing adversities and self-harm behaviours. These findings are congruent with diathesis–stress models of suicide risk that underscore the importance of having trusted others to rely on in the presence of near-term strain, worry and adversity,^6,7^ and suggest that public health campaigns that promote an increase in practical forms of support may help reduce suicide risk.^3^ These findings highlight the need for relatives, friends and neighbours to be encouraged to reach out to others who may be experiencing COVID-19 hardships such as unemployment, accessing essential items such as food or medicine, or contracting the virus itself.^3,41^

### Strengths and limitations

This study has a number of strengths as well as limitations. Strengths of this study include a long follow-up period with repeated measurements of predictor and outcome variables and the use of a large, well-stratified sample on key demographic groups. Although data were weighted on the basis of population estimates of core demographics, sampling was not random, and the findings can therefore not be generalised to the UK population as a whole. However, our goal was to identify associations between predictors and outcomes, and not to present population prevalence estimates. We also used a statistical modelling approach that accounted for time-invariant risk factors for self-harm such as genetic predisposition and adversity early in life^6,7^ and is thus an improvement upon prior research that did not account for these factors.

This study also has several limitations. First, there were some differences in the wording of our measures of adversities and worries about adversities (see Supplementary Table 3), and although selected to be congruent with one another, they may not therefore have captured the exact same adversity and worry. Second, our measures of face-to-face/telephone/video contact lacked detail on who the participant was speaking with, and prior work from our research group suggests significant variability in the types of contacts accessed by people who report self-harm thoughts and behaviours.^15^ Third, our measure of self-harm behaviours did not specify what self-harming was and participants may therefore have not reported behaviours they did not consider to be self-harming, but which may clinically be considered as such (for example self-poisoning or intentional destruction of bodily tissue). Our measure of self-harm behaviours also prompted respondents to report how often they had been bothered by self-harm, which may have led to underreporting given that one of the most common reasons for self-harm is to mitigate and soothe distress.^25^ Fourth, we analysed data across 15 months, which included three different lockdowns when social support was largely provided virtually. It therefore remains unclear whether there were differences in the associations between the social factors we examined and outcomes depending on the precise social restrictions in place. Finally, because fixed-effects analysis does not address the direction of the relationship between exposures and outcomes, we were not able to establish temporal precedence, so it is possible that self-harming led to changes in social behaviours. Nonetheless, our findings highlight the relationship between these factors.

### Implications

Our results demonstrate the importance of loneliness and social support for individuals during a pandemic, especially those who are facing adversities, highlighting their associations with self-harm thoughts and behaviours. Although modest, our moderation findings suggest that social support and loneliness help to buffer and exacerbate, respectively, against adversities. The provision of social support could therefore help to reduce the impact of pandemic-related adversities on self-harm. Although this study does not focus on suicide rates, self-harming is a strong risk factor for suicide risk, so helping to reduce risk factors for self-harming is an important mitigation strategy.^2,41^ It is therefore critical that policymakers and public health leaders not only focus on reducing adversities such as those relating to employment and financial hardship during the COVID-19 pandemic and potential future pandemics, but also develop community schemes that help to reduce loneliness and increase social support as part of self-harm and suicide prevention strategies. This is particularly important even as the pandemic abates as the detrimental impact of the pandemic on self-harm and suicide is likely to accumulate and may even peak after the actual pandemic is under control.^4^

## Data Availability

The code used to run the analysis is available at https://osf.io/hmn9s/.

## Supplementary material

For supplementary material accompanying this paper visit http://dx.doi.org/10.1192/bjo.2021.1071. click here to view supplementary material

## References

[1] Gunnell D, Appleby L, Arensman E, Hawton K, John A, Kapur N, Suicide risk and prevention during the COVID-19 pandemic. Lancet Psychiatry 2020; 7: 468–71. 32330430 10.1016/S2215-0366(20)30171-1 PMC7173821

[2] Wasserman D, Iosue M, Wuestefeld A, Carli V. Adaptation of evidence-based suicide prevention strategies during and after the COVID-19 pandemic. World Psychiatry 2020; 19: 294–306. 32931107 10.1002/wps.20801 PMC7491639

[3] Moutier C. Suicide prevention in the COVID-19 era: transforming threat into opportunity. JAMA Psychiatry 2021; 78(4): 433–8. 10.1001/jamapsychiatry.2020.3746 33064124

[4] Sher L. The impact of the COVID-19 pandemic on suicide rates. QJM Int J Med 2020; 113: 707–12. 10.1093/qjmed/hcaa202 PMC7313777 32539153

[5] Brodsky BS. Early childhood environment and genetic interactions: the diathesis for suicidal behavior. Curr Psychiatry Rep 2016; 18: 86. 27484207 10.1007/s11920-016-0716-z

[6] O'Connor RC, Nock MK. The psychology of suicidal behaviour. Lancet Psychiatry 2014; 1: 73–85. 26360404 10.1016/S2215-0366(14)70222-6

[7] Turecki G, Brent DA, Gunnell D, O'Connor RC, Oquendo MA, Pirkis J, Suicide and suicide risk. Nat Rev Dis Primer 2019; 5: 1–22. 10.1038/s41572-019-0121-0 31649257

[8] Beckman K, Mittendorfer-Rutz E, Lichtenstein P, Larsson H, Almqvist C, Runeson B, Mental illness and suicide after self-harm among young adults: Long-term follow-up of self-harm patients, admitted to hospital care, in a national cohort. Psychol Med 2016; 46: 3397–405. 27644850 10.1017/S0033291716002282

[9] Hawton K, Bergen H, Cooper J, Turnbull P, Waters K, Ness J, Suicide following self-harm: findings from the multicentre study of self-harm in England, 2000–2012. J Affect Disord 2015; 175: 147–51. 25617686 10.1016/j.jad.2014.12.062

[10] Carr MJ, Steeg S, Webb RT, Kapur N, Chew-Graham CA, Abel KM, Effects of the COVID-19 pandemic on primary care-recorded mental illness and self-harm episodes in the UK: a population-based cohort study. Lancet Public Health 2021; 6: e124–35. 33444560 10.1016/S2468-2667(20)30288-7 PMC7843955

[11] Jollant F, Roussot A, Corruble E, Chauvet-Gelinier J-C, Falissard B, Mikaeloff Y, Hospitalization for self-harm during the early months of the COVID-19 pandemic in France: a nationwide retrospective observational cohort study. Lancet Reg Health Eur 2021; 6: 100102. 34557830 10.1016/j.lanepe.2021.100102 PMC8454825

[12] Hawton K, Casey D, Bale E, Brand F, Ness J, Waters K, Self-harm during the early period of the COVID-19 pandemic in England: comparative trend analysis of hospital presentations. J Affect Disord 2021; 282: 991–5. 33601744 10.1016/j.jad.2021.01.015 PMC7832687

[13] Steeg S, Bojanić L, Tilston G, Williams R, Jenkins DA, Carr MJ, Temporal trends in primary care-recorded self-harm during and beyond the first year of the COVID-19 pandemic: time series analysis of electronic healthcare records for 2.8 million patients in the greater manchester care record. EClinicalMedicine 2021; 41: 101175. 34746726 10.1016/j.eclinm.2021.101175 PMC8557994

[14] Centers for Disease Control and Prevention (CDC). Mental Health, Substance Use, and Suicidal Ideation During the COVID-19 Pandemic — United States, June 24–30, 2020. National Center for Injury Prevention and Control, 2020 (https://www.ncbi.nlm.nih.gov/pmc/articles/PMC7440121/).

[15] Iob E, Steptoe A, Fancourt D. Abuse, self-harm and suicidal ideation in the UK during the COVID-19 pandemic. Br J Psychiatry 2020; 217: 543–6. 32654678 10.1192/bjp.2020.130 PMC7360935

[16] O'Connor RC, Wetherall K, Cleare S, McClelland H, Melson AJ, Niedzwiedz CL, Mental health and well-being during the COVID-19 pandemic: longitudinal analyses of adults in the UK COVID-19 Mental Health & Wellbeing Study. Br J Psychiatry 2021; 218: 326–33. 10.1192/bjp.2020.212 PMC7684009 33081860

[17] Dubé JP, Smith MM, Sherry SB, Hewitt PL, Stewart SH. Suicide behaviors during the COVID-19 pandemic: a meta-analysis of 54 studies. Psychiatry Res 2021; 301: 113998. 34022657 10.1016/j.psychres.2021.113998 PMC9225823

[18] Leslie E, Wilson R. Sheltering in place and domestic violence: evidence from calls for service during COVID-19. J Public Econ 2020; 189: 104241. 32834179 10.1016/j.jpubeco.2020.104241 PMC7377795

[19] International Labour Organization. ILO Monitor: COVID-19 and the World of Work (7th edn). International Labour Organization, 2021 (https://www.ilo.org/wcmsp5/groups/public/@dgreports/@dcomm/documents/briefingnote/wcms_767028.pdf).

[20] Office for National Statistics. Labour Market Overview. ONS, 2020 (https://www.ons.gov.uk/employmentandlabourmarket/peopleinwork/employmentandemployeetypes/bulletins/uklabourmarket/december2020).

[21] Paul E, Fancourt D. Factors influencing self-harm thoughts and behaviours over the first year of the COVID-19 pandemic in the UK: longitudinal analysis of 49 324 adults. Br J Psychiatry [Epub ahead of print] 14 Sept 2021; Available from: 10.1192/bjp.2021.130. PMC8958127 35045899

[22] Mackin DM, Perlman G, Davila J, Kotov R, Klein DN. Social support buffers the effect of interpersonal life stress on suicidal ideation and self-injury during adolescence. Psychol Med 2017; 47: 1149–61. 27995812 10.1017/S0033291716003275

[23] Tham S-G, Ibrahim S, Hunt IM, Kapur N, Gooding P. Examining the mechanisms by which adverse life events affect having a history of self-harm, and the protective effect of social support. J Affect Disord 2020; 263: 621–8. 31744741 10.1016/j.jad.2019.11.037

[24] Doyle L, Sheridan A, Treacy MP. Motivations for adolescent self-harm and the implications for mental health nurses. J Psychiatr Ment Health Nurs 2017; 24: 134–42. 28124465 10.1111/jpm.12360

[25] Edmondson AJ, Brennan CA, House AO. Non-suicidal reasons for self-harm: a systematic review of self-reported accounts. J Affect Disord 2016; 191: 109–17. 26655120 10.1016/j.jad.2015.11.043

[26] Peplau LA, Perlman D. Perspectives on loneliness. In Loneliness: A Sourcebook of Current Theory, Research and Practice (eds LA Peplau, D Perlman): 1–17. John Wiley, 1982.

[27] Durkheim E. A Study in Sociology. Routledge & K. Paul London, 1952.

[28] McGinty EE, Presskreischer R, Han H, Barry CL. Psychological distress and loneliness reported by US adults in 2018 and April 2020. JAMA 2020; 324(1): 93–4. 32492088 10.1001/jama.2020.9740 PMC7270868

[29] Office for National Statistics. Mapping Loneliness During the Coronavirus Pandemic. ONS, 2021 (https://www.ons.gov.uk/peoplepopulationandcommunity/wellbeing/articles/mappinglonelinessduringthecoronaviruspandemic/2021-04-07).

[30] Allison P. Fixed Effects Regression Models. SAGE Publications, 2009 (http://methods.sagepub.com/book/fixed-effects-regression-models).

[31] Hawton K, Harriss L, Hall S, Simkin S, Bale E, Bond A. Deliberate self-harm in Oxford, 1990–2000: a time of change in patient characteristics. Psychol Med 2003; 33: 987–95. 12946083 10.1017/s0033291703007943

[32] National Collaborating Centre for Mental Health (Great Britain), National Institute for Health and Clinical Excellence (Great Britain), British Psychological Society. Self-Harm: Longer-Term Management. National Clinical Guidelines. NICE, 2012 (https://www.ncbi.nlm.nih.gov/books/NBK126777).

[33] Löwe B, Kroenke K, Herzog W, Gräfe K. Measuring depression outcome with a brief self-report instrument: sensitivity to change of the patient health questionnaire (PHQ-9). J Affect Disord 2004; 81: 61–6. 15183601 10.1016/S0165-0327(03)00198-8

[34] Kliem S, Mößle T, Rehbein F, Hellmann DF, Zenger M, Brähler E. A brief form of the perceived social support questionnaire (F-SozU) was developed, validated, and standardized. J Clin Epidemiol 2015; 68: 551–62. 25499982 10.1016/j.jclinepi.2014.11.003

[35] Lin M, Hirschfeld G, Margraf J. Brief form of the perceived social support questionnaire (F-SozU K-6): validation, norms, and cross-cultural measurement invariance in the USA, Germany, Russia, and China. Psychol Assess 2019; 31: 609. 30589275 10.1037/pas0000686

[36] Russell D, Peplau LA, Cutrona CE. The revised UCLA Loneliness Scale: Concurrent and discriminant validity evidence. J Pers Soc Psychol 1980; 39(3): 472–80. 7431205 10.1037//0022-3514.39.3.472

[37] Allison PD. Fixed Effects Regression Models. SAGE publications, 2009.

[38] Office for National Statistics. Population Estimates for the UK, England and Wales, Scotland and Northern Ireland. ONS, 2020 (https://www.ons.gov.uk/peoplepopulationandcommunity/populationandmigration/populationestimates/bulletins/annualmidyearpopulationestimates/mid2018).

[39] Hainmueller J, Xu Y. Ebalance: a stata package for entropy balancing. J Stat Softw 2013; 54: 1–18.

[40] StataCorp. Stata Statistical Software: Release 16. StataCorp LP, 2019.

[41] Niederkrotenthaler T, Gunnell D, Arensman E, Pirkis J, Appleby L, Hawton K, Suicide Research, Prevention, and COVID-19. Hogrefe Publishing, 2020. 10.1027/0227-5910/a000731 PMC8729451 32716205

[42] Wright L, Steptoe A, Fancourt D. Does thinking make it so? Differential associations between adversity worries and experiences and mental health during the COVID-19 pandemic. J Epidemiol Community Health 2021; 75: 817–23. 33483341 10.1136/jech-2020-215598 PMC7830321

