# Supplementary material for: The interaction between social factors and adversities on self-harm during the COVID-19 pandemic: longitudinal analysis of 49 227 UK adults
Source: BJPsych Open. 2021 Dec 10;8(1):e12. doi: 10.1192/bjo.2021.1071 (PMC8674193; doi:10.1192/bjo.2021.1071)
Supplement: Supplementary file 1 [file S2056472421010711sup001.docx]

Supplemental Materials

Statistical Analysis

The basic fixed effects regression model can be expressed as follows:

Outcome_𝑖𝑡_= 𝛽_0𝑡_+ 𝛽_1_𝐸𝑖𝑘𝑡 + 𝛽2𝐷𝑡 + 𝛽3𝑁𝑡 + 𝛼𝑖 + 𝜀𝑖𝑡

where Outcome*_it_* is a measure of individual 𝑖's self-harm thoughts or self-harm behaviours at time 𝑡, E is individual 𝑖’s predictor variable at time 𝑡, D𝑡 is a vector of indicator variables for day, *N𝑡* is a continuous variable for days since lockdown, is unobserved time invariant confounding factors, and 𝜀 is error.

Table S1: Comparison of included and excluded participants, weighted

|  |  | Included  n = 49,227 | | Excluded  n = 17,081 | | Exclusion | | |
| --- | --- | --- | --- | --- | --- | --- | --- | --- |
| Variable |  | M | SD | M | SD | OR | 95% CI | |
| Gender | Female (ref male) | 0.75 | 0.43 | 0.73 | 0.44 | 0.91* | 0.88 | 0.94 |
| Age | 18-29 | 0.07 | 0.26 | 0.17 | 0.37 | ref. |  |  |
|  | 30-44 | 0.25 | 0.43 | 0.35 | 0.48 | 0.56* | 0.53 | 0.59 |
|  | 45-59 | 0.35 | 0.48 | 0.29 | 0.45 | 0.30* | 0.29 | 0.32 |
|  | 60+ | 0.33 | 0.47 | 0.18 | 0.39 | 0.18* | 0.17 | 0.19 |
| Ethnicity | Ethnic minority groups (ref white) | 0.05 | 0.21 | 0.09 | 0.28 | 2.29* | 2.18 | 2.41 |
| Income | £90k+ | 0.11 | 0.31 | 0.11 | 0.31 | ref. |  |  |
|  | £60k - £90k | 0.16 | 0.36 | 0.15 | 0.36 | 0.90* | 0.83 | 0.98 |
|  | £30k - £60k | 0.35 | 0.48 | 0.33 | 0.47 | 0.88* | 0.82 | 0.94 |
|  | £16k - £30k | 0.24 | 0.43 | 0.24 | 0.43 | 0.99* | 0.92 | 1.06 |
|  | <£16k | 0.14 | 0.35 | 0.18 | 0.38 | 1.20* | 1.12 | 1.30 |
| Education | Up to GCSE | 0.13 | 0.34 | 0.18 | 0.39 | ref. |  |  |
|  | A-levels or vocational | 0.17 | 0.38 | 0.20 | 0.40 | 0.96 | 0.92 | 1.00 |
|  | Undergraduate | 0.41 | 0.49 | 0.38 | 0.48 | 0.69* | 0.65 | 0.72 |
|  | Postgraduate | 0.28 | 0.45 | 0.24 | 0.43 | 0.65* | 0.61 | 0.69 |
| Long-Term Physical Health Condition | Yes (ref no) | 0.40 | 0.49 | 0.36 | 0.48 | 0.70* | 0.67 | 0.72 |
| Long-Term Mental Health Condition | Yes (ref no) | 0.18 | 0.39 | 0.23 | 0.42 | 1.34* | 1.29 | 1.40 |
| Country | England | 0.81 | 0.39 | 0.83 | 0.38 | ref |  |  |
|  | Wales | 0.12 | 0.32 | 0.09 | 0.29 | 0.67* | 0.61 | 0.73 |
|  | Scotland | 0.06 | 0.24 | 0.07 | 0.25 | 1.16* | 1.09 | 1.24 |
|  | Northern Ireland | 0.01 | 0.10 | 0.01 | 0.11 | 1.96* | 1.77 | 2.16 |
| Self-Harm Thoughts | | 0.10 | 0.30 | 0.15 | 0.36 | 1.85* | 1.76 | 1.93 |
| Self-Harm Behaviours | | 0.02 | 0.15 | 0.05 | 0.21 | 2.64* | 2.44 | 2.85 |
| Adversity Experiences | | 1.44 | 0.65 | 1.55 | 0.76 | 1.30* | 1.27 | 1.33 |
| Adversity Worries | | 2.37 | 1.30 | 2.33 | 1.39 | 0.95* | 0.94 | 0.97 |
| Social Support | | 3.74 | 1.01 | 3.65 | 1.04 | 0.90* | 0.88 | 0.91 |
| Emotional Support | | 3.82 | 1.06 | 3.69 | 1.10 | 0.88* | 0.86 | 0.89 |
| Instrumental Support | | 3.66 | 1.15 | 3.61 | 1.16 | 0.95* | 0.94 | 0.96 |
| Loneliness | | 1.62 | 0.63 | 1.73 | 0.66 | 1.40* | 1.37 | 1.44 |
| Face-to-face Contact | | 5.25 | 2.80 | 5.20 | 2.75 | 0.99* | 0.98 | 0.99 |
| Telephone/Video Contact | | 4.23 | 2.34 | 4.20 | 2.41 | 1.01* | 1.00 | 1.02 |

Note. * indicates p < .05. Data were weighted to the proportions of gender, age, ethnicity, country, and education obtained from the Office for National Statistics

# Measures

## Social Support

### Perceived Social Support

Social support in the past week was measured using an adapted version of the six-item short form of Perceived Social Support Questionnaire (F-SozU K-6).^35,36^ Each item is rated on a 5-point scale from “not true at all” to “very true”, with higher scores indicating higher levels of perceived social support. Minor adaptations made to question phrasing to make it relevant to experiences during COVID-19 can be found in Table S2.

Table S2: Adapted Perceived Social Support Questionnaire (F-SozU K-6)

| Original | Study-adapted |
| --- | --- |
| I experience a lot of understanding and security from others | I have experienced a lot of understanding and support from others (emotional support) |
| I know a very close person whose help I can always count on | I have a very close person whose help I can always count on (emotional support) |
| If necessary, I can easily borrow something I might need from neighbours or friends | If necessary, I can easily borrow something I need from neighbours or friends (instrumental support) |
| I know several people with whom I like to do things | I have people with whom I can spend time and do things together (instrumental support) |
| When I am sick, I can without hesitation ask friends and family to take care of  important matters for me | If I get sick, I have friends and family who will take care of me (instrumental support) |
| If I am down, I know to whom I can go to without hesitation | If I am feeling down, I have people I can talk to without hesitation (emotional support) |

## Adversity experiences and adversity worries

Five categories of adversities were considered: finances, COVID-19 illness, social relationships, personal safety, and accessing essentials. We used multiple questionnaire items to measure each category (Table S4). Categories were treated as binary (absent vs. present) and were summed to create an index of the number of adversities experiences or worries at each time point. Adversity worries were captured from two multiple choice questions asked each wave: “Over the past week, have any of the following been worrying you at all, even if only in a minor way?”; “Have any of these things been causing you SIGNIFICANT stress? (e.g., they have been constantly on your mind or have been keeping you awake at night)”. The same response categories were given for each question.

Table S3: Questionnaire items used to measure adversity experiences and worries

| Type of adversity | Adversity experiences | Adversity worries |
| --- | --- | --- |
| Financial | Lost your job/been unable to do paid work  Your spouse/partner lost their job or was unable to do paid work  Unable to pay bills/ rent/ mortgage  Major cut in household income (e.g., due to you or your partner being furloughed/ put on leave/ not receiving sufficient work)  Evicted/lost accommodation | Work (even if you feel your job is safe)  Losing your job / unemployment  Finances |
| COVID-19 illness | Currently have or previously had suspected or diagnosed COVID-19 | Catching COVID-19  Becoming seriously ill from COVID-19 |
| Social and relationship concerns | Somebody close to you is ill in hospital (due to COVID-19 or another illness)  You lost somebody close to you (due to COVID-19 or another cause) | Marriage or other romantic relationship  Friends or family living in your household  Friends or family living outside your household  Neighbours  Your pet |
| Accessing essentials | Unable to access sufficient food  Unable to access required medication | Getting medication  Getting food |
| Personal safety | Being physically harmed or hurt by somebody else  Being bullied, controlled, intimidated, or psychologically hurt by someone else | Your own safety / security |

## Variables used to describe the sample

All demographic and socio-economic variables were measured at baseline interview: country of residence (England, Scotland, Wales, Northern Ireland), gender (male, female), ethnicity (White, ethnic minority groups), age (18-29, 30-44, 45-59, 60+), annual income (< £16k, £16k - £30k, £30k - £60k, £60k - £90k, £90k +), and education level (up to GCSE, A-levels or equivalent, undergraduate degree, postgraduate degree).

Long-term physical health condition (yes, no) was assessed using a multiple-choice question on medical conditions. Included conditions were high blood pressure, diabetes, heart disease, lung disease, cancer, any other clinically diagnosed chronic physical health conditions, or any disability. Long-term mental health condition (yes, no) was assessed with the same multiple choice question using items on clinically diagnosed depression, clinically diagnosed anxiety, and any other clinically diagnosed mental health problem.

Table S4: Descriptive statistics for entire sample and for those with variation in each outcome, unweighted and weighted

|  |  | Total sample  n = 49,227 | | Total sample  n = 49,227 | | Sample with variation in self-harm thoughts  n = 11,559 | | Sample with variation in self-harm behaviours  n = 3,740 | |
| --- | --- | --- | --- | --- | --- | --- | --- | --- | --- |
|  |  | unweighted | | weighted | | weighted | | weighted | |
| Variable |  | M | SD | M | SD | M | SD | M | SD |
| Gender | Female | 0.75 | 0.43 | 0.52 | 0.50 | 0.55 | 0.50 | 0.55 | 0.50 |
| Age | 18-29 | 0.07 | 0.26 | 0.13 | 0.33 | 0.18 | 0.38 | 0.23 | 0.42 |
|  | 30-44 | 0.25 | 0.43 | 0.21 | 0.41 | 0.25 | 0.43 | 0.25 | 0.43 |
|  | 45-59 | 0.35 | 0.48 | 0.29 | 0.45 | 0.29 | 0.46 | 0.30 | 0.46 |
|  | 60+ | 0.33 | 0.47 | 0.38 | 0.49 | 0.28 | 0.45 | 0.21 | 0.41 |
| Ethnicity | Ethnic minority groups | 0.05 | 0.21 | 0.09 | 0.29 | 0.12 | 0.33 | 0.13 | 0.34 |
| Income | £90k+ | 0.11 | 0.31 | 0.08 | 0.27 | 0.06 | 0.24 | 0.05 | 0.22 |
|  | £60k - £90k | 0.16 | 0.36 | 0.12 | 0.33 | 0.11 | 0.31 | 0.08 | 0.27 |
|  | £30k - £60k | 0.35 | 0.48 | 0.33 | 0.47 | 0.30 | 0.46 | 0.26 | 0.44 |
|  | £16k - £30k | 0.24 | 0.43 | 0.28 | 0.45 | 0.28 | 0.45 | 0.26 | 0.44 |
|  | <£16k | 0.14 | 0.35 | 0.20 | 0.40 | 0.24 | 0.43 | 0.35 | 0.48 |
| Education | Up to GCSE | 0.13 | 0.34 | 0.31 | 0.46 | 0.30 | 0.46 | 0.33 | 0.47 |
|  | A-levels or vocational | 0.17 | 0.38 | 0.33 | 0.47 | 0.35 | 0.48 | 0.36 | 0.48 |
|  | Undergraduate | 0.41 | 0.49 | 0.22 | 0.41 | 0.21 | 0.40 | 0.18 | 0.39 |
|  | Postgraduate | 0.28 | 0.45 | 0.15 | 0.35 | 0.15 | 0.36 | 0.12 | 0.33 |
| Long-Term Physical Health Condition | Yes | 0.40 | 0.49 | 0.44 | 0.50 | 0.48 | 0.50 | 0.53 | 0.50 |
| Long-Term Mental Health Condition | Yes | 0.18 | 0.39 | 0.19 | 0.39 | 0.34 | 0.48 | 0.52 | 0.50 |
| Country | England | 0.81 | 0.39 | 0.84 | 0.36 | 0.85 | 0.36 | 0.84 | 0.36 |
|  | Wales | 0.12 | 0.32 | 0.06 | 0.23 | 0.05 | 0.22 | 0.05 | 0.23 |
|  | Scotland | 0.06 | 0.24 | 0.08 | 0.27 | 0.09 | 0.28 | 0.09 | 0.28 |
|  | Northern Ireland | 0.01 | 0.10 | 0.02 | 0.14 | 0.02 | 0.12 | 0.02 | 0.13 |

Note. Data were weighted to the proportions of gender, age, ethnicity, country, and education obtained from the Office for National Statistics.

Table S5: Sensitivity analysis: associations between disaggregated social support with self-harm thoughts and behaviours (main effects) derived from fixed effects logistic regression models

|  | Self-Harm Thoughts | | | Self-Harm Behaviours | | |
| --- | --- | --- | --- | --- | --- | --- |
| Variable | OR | 95% CI | | OR | 95% CI | |
| Emotional Support | 0.53* | 0.51 | 0.55 | 0.79* | 0.74 | 0.83 |
| Instrumental Support | 0.76* | 0.74 | 0.79 | 0.77* | 0.72 | 0.81 |

Note. * indicates p < .05. Data were weighted to the proportions of gender, age, ethnicity, country, and education obtained from the Office for National Statistics

Table S6: Sensitivity analyses: associations between adversity and worries with self-harm thoughts and behaviours (main effects and interaction terms with the social support variable disaggregated) derived from fixed effects logistic regression models

|  | Self-Harm Thoughts | | | Self-Harm Behaviours | | |
| --- | --- | --- | --- | --- | --- | --- |
| Variable | OR | 95% CI | | OR | 95% CI | |
| **ADVERSITY EXPERIENCES** | 1.15* | 1.13 | 1.17 | 1.12* | 1.08 | 1.15 |
| Emotional Support | 0.47* | 0.46 | 0.48 | 0.69* | 0.66 | 0.73 |
| Interaction w/Emotional Support | 1.02* | 1.00 | 1.03 | 0.99 | 0.97 | 1.01 |
| Adversity Experiences | 1.12* | 1.10 | 1.14 | 1.08 | 1.05 | 1.12 |
| Instrumental Support | 0.55* | 0.53 | 0.56 | 0.68* | 0.65 | 0.72 |
| Interaction w/Instrumental Support | 0.99 | 0.98 | 1.00 | 0.96* | 0.94 | 0.98 |
| **ADVERSITY WORRIES** | 1.43* | 1.39 | 1.46 | 1.33* | 1.27 | 1.38 |
| Emotional Support | 0.47* | 0.46 | 0.48 | 0.68* | 0.65 | 0.71 |
| Interaction w/Emotional Support | 1.02* | 1.00 | 1.04 | 1.04* | 1.01 | 1.07 |
| Adversity Worries | 1.40* | 1.37 | 1.43 | 1.30* | 1.24 | 1.35 |
| Instrumental Support | 0.55* | 0.54 | 0.57 | 0.67* | 0.64 | 0.71 |
| Interaction w/Instrumental Support | 1.01 | 0.99 | 1.03 | 1.02 | 0.99 | 1.05 |

*Note. * indicates p < .05. Data were weighted to the proportions of gender, age, ethnicity, country, and education obtained from the Office for National Statistics.*
